# Targeted Therapy in Cardiovascular Disease: A Precision Therapy Era

**DOI:** 10.3389/fphar.2021.623674

**Published:** 2021-04-16

**Authors:** Mengda Xu, Kailun Zhang, Jiangping Song

**Affiliations:** ^1^ Union Hospital, Tongji Medical College, Huazhong University of Science and Technology, Wuhan, China; ^2^ State Key Laboratory of Cardiovascular Disease, Fuwai Hospital, National Center for Cardiovascular Diseases, Chinese Academy of Medical Sciences and Peking Union Medical College, Beijing, China

**Keywords:** targeted therapy, antibody, gene editing, nucleic acid drugs, cell therapy, cardiovascular disease

## Abstract

Targeted therapy refers to exploiting the specific therapeutic drugs against the pathogenic molecules (a protein or a gene) or cells. The drug specifically binds to disease-causing molecules or cells without affecting normal tissue, thus enabling personalized and precision treatment. Initially, therapeutic drugs included antibodies and small molecules, (e.g. nucleic acid drugs). With the advancement of the biology technology and immunotherapy, the gene editing and cell editing techniques are utilized for the disease treatment. Currently, targeted therapies applied to treat cardiovascular diseases (CVDs) mainly include protein drugs, gene editing technologies, nucleic acid drugs and cell therapy. Although targeted therapy has demonstrated excellent efficacy in pre-clinical and clinical trials, several limitations need to be recognized and overcome in clinical application, (e.g. off-target events, gene mutations, etc.). This review introduces the mechanisms of different targeted therapies, and mainly describes the targeted therapy applied in the CVDs. Furthermore, we made comparative analysis to clarify the advantages and disadvantages of different targeted therapies. This overview is expected to provide a new concept to the treatment of the CVDs.

## Introduction

Cardiovascular diseases are the leading cause of death worldwide (Ghantous et al., 2020). CVDs are a broad spectrum of diseases that can be classified into different categories based on different criteria. For example, congenital heart disease and acquired heart disease are classified according to the time of onset. The etiology of CVDs is complex, including metabolic abnormalities, genetic alterations, abnormal protein function and other factors (Tanai and Frantz, 2015). All CVDs eventually progress to heart failure (HF) if not effectively treated. HF affects 1–2% of the world’s population and places a heavy burden on the society (Tanai and Frantz, 2015). Current treatments for CVDs mainly include traditional pharmacotherapy and surgery (Stehlik et al., 2018; Felker et al., 2020; Zelniker and Braunwald, 2020). Although the above methods alleviate the symptoms of the disease and reduce the mortality rate, both methods have certain drawbacks. Traditional medication is less invasive, but it can cause damage to the liver, kidneys and other organs, as well as other side effects (Lassiter et al., 2020). In spite of the excellent effectiveness, the clinical application of cardiac surgery is always restrained by the complex procedures and the possibility of postoperative complications (Roth et al., 2020). Therefore, there is an urgent and unmet need to develop a novel, convenient, and efficient approach for the treatment of CVDs.

The success of the human genome project and the rapid development of molecular biology facilitate the precise detection of genome, transcriptome and proteome changes. By using these methods, researchers can explore the mechanisms underlying the progression of diseases and design new drugs that target to the pathogenic molecules, which is named targeted therapy (Bedard et al., 2020). By specifically targeting and binding to abnormal genes or proteins, the novel regimen enables personalized and efficient therapy (Tsimberidou et al., 2014). Over the past years, the striking breakthroughs of the gene editing and cell therapy techniques have led the targeted therapy to a vigorous development stage. To date, a variety of drugs have been utilized to treat cancers, such as trastuzumab in breast tumor and chimeric antigen receptor-modified T (CAR-T) in hematological malignancies and have demonstrated considerable effectiveness (Nicolazzi et al., 2018; Zhao et al., 2018a).

In addition to the application in cancers, targeted therapy is also playing an important role in the treatment of CVDs. Some CVDs are caused by the gene mutation, (e.g. *MYH6* in hypertrophic cardiomyopathy (HCM)) (Mosqueira et al., 2018) or an abnormal protein, (e.g. fibroblast activation protein (FAP) in the cardiac fibrosis), which provides a rationale for the targeted therapy in CVDs (Aghajanian et al., 2019). Actually, increasing targeted therapies have been used to treat some CVDs and have exhibited promising effect, such as evolocumab (a type of monoclonal antibody (mAb)) in the treatment of homozygous familial hypercholesterolemia (HoFH). Herein, this review introduced the mechanisms of various targeted therapies, and depicted the landscape of targeted therapy applied in CVDs. Furthermore, a comparative analysis was performed to clarify both the advantages and limitations of the applications of targeted therapies in CVDs.

## Protein

### Antibodies

Antibodies can specifically recognize and bind to the epitopes of the antigen. Based on the number of binding epitopes, antibodies used for targeted therapies can be classified as mAbs or bispecific antibodies (bAbs). Here, we summarize the mechanisms and applications of the two types of antibodies.

### mAbs

**FIGURE 1:**
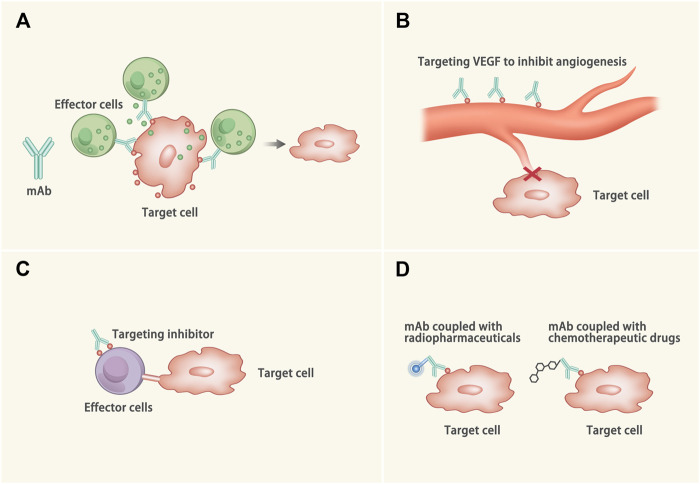
Mechanisms of the mAb. **(A)** The Fab of the mAb binds to the target epitope and the Fc of the mAb binds to the effector cell (such as the natural killer cell) or the complement to kill the target cells through antibody-dependent cell-mediated cytotoxicity, complement-mediated cytotoxicity or directly inhibit abnormal signals of the target cells. **(B)** The mAb binds to the growth factor (such as VEGF) to inhibit the angiogenesis of the target cells. **(C)** The interaction between some ligands and receptors (such as PD-1/PD-L1) can inactivate the effector cells. The mAb binds to the inhibitory molecule to protect the effector cells from dysfunction. **(D)** The mAbs are equipped with radiopharmaceuticals or chemotherapeutic drugs. When the mAbs binds to the target cells, the drugs come close to the target cell and kill the target cells.

At present, mAbs have been widely applied in malignancies and rheumatic diseases. And mAbs have been exploited to treat CVDs (Smyth, 2017; Schmid and Neri, 2019). mAbs exert the therapeutic efficacy via the following four ways (Figure 1): 1) Activating immune response to the abnormal tissues: Once binding to the target epitope, mAbs can mediate antibody-dependent cellular cytotoxicity, complement-mediated cytotoxicity or directly inhibit abnormal signals of target cells, (e.g. alemtyzymab) (Kennedy and Hillmen, 2002). 2) Inhibiting survival of the pathogenic tissues: mAbs can bind to the growth factors and block the angiogenesis of the lesioned tissues, (e.g. bevacizumab) (Chellappan et al., 2018). 3) Blocking inhibitory signals of the effector cells: The interaction between the programmed cell death protein 1 (PD-1) receptor and its ligand (PD-L1) results in T cells dysfunction, which could be retrieved by certain mAbs via blocking the PD-1/PD-L1 signal, (e.g. nivolumab) (Ding et al., 2019). 4) Coupling with the therapeutic drugs: The mAbs equipped with radiopharmaceuticals or chemotherapeutic drugs could help to deliver and release drugs after binding to the target molecules, (e.g. Ado-trastuzumab emtansine) (García-Alonso et al., 2020).

Possessing the above characteristics, mAbs have also shown excellent efficacy in the cardiovascular field. Gain-of-function mutations (*p*.S127R, *p*. F216L and *p*. D374Y) of *proprotein convertase subtilisin/kexin type 9* (*PCSK9*) can lead to autosomal dominant hypercholesterolemia (by reducing low-density lipoprotein receptor (LDLR) levels) and increase the susceptibility to CVDs (Abifadel et al., 2007; Homer et al., 2008). As the first PCSK9 inhibitor approved by the Food and Drug Administration (FDA), the alirocumab was used in the ODYSSEY OUTCOMES trial and its effect on cardiovascular mortality after acute coronary syndromes (ACS) was examined (Schwartz et al., 2018). The trial included 18,924 patients who suffered from an ACS 1–12 months earlier. All patients had a high level of LDL cholesterol (LDL-C) (>1.8 mmol/L), non-high-density lipoprotein cholesterol (non-HDL-C) level (>2.6 mmol/L) or apolipoprotein B (>80 mg/dl) after receiving a high-intensity dose or the maximum tolerated dose of statin. Patients were randomly assigned to receive alirocumab (*n* = 9,462, 75 mg every 2 weeks) or placebo (*n* = 9,462). During the study, the dose of alirocumab was adjusted to maintain the cholesterol level at 0.65–1.30 mmol/L. The primary endpoint was a composite of death from fatal or nonfatal ischemic stroke, nonfatal myocardial infarction (MI), coronary heart disease (CHD), or unstable angina (UA) requiring hospitalization. After 2.8 years of follow-up, the results showed that the alirocumab could reduce the incidence of the primary endpoint (hazard ratio [HR], 0.85; 95% confidence interval [CI], 0.78 to 0.93; *p* < 0.001). Patients with a higher baseline LDL-C level(> 100 mg/dl) gained more benefits than patients with a lower baseline LDL-C level. Besides, evolocumab is another mAb that inhibits PCSK9. Binding of the evolocumab to PCSK9 resulted in a rise in hepatic surface LDLR and an increase in plasma LDL-C clearance (Sabatine et al., 2017a). In the FOURIER study, 27,564 patients with LDL-C higher than 70 mg/dl after statin treatment were randomly assigned (1:1) to receive evolocumab (140 mg/2 weeks or 420 mg/month) or placebo (Sabatine et al., 2017b). The primary endpoint was a composite of cardiovascular death, UA requiring hospitalization, MI, stroke, or coronary revascularization. After a median follow-up of 2.2 years, the results demonstrated that the evolocumab significantly reduced the risk of cardiovascular events in patients with (HR, 0.83; 95% CI, 0.75 to 0.93; *p* = 0.0008) and without (HR, 0.87; 95% CI, 0.79 to 0.96; *p* = 0.0052) diabetes. However, the long-term efficacy of the evolocumab on high LDL-C needs to be evaluated in further studies with longer follow-up.

Angiopoietin-like protein 3 (ANGPTL3) can inhibit the activity of lipoprotein lipase and increase the content of triglyceride and other lipids in plasma (Ono et al., 2003). Loss-of-function mutation of *ANGPTL3* has been found to relate to lower levels of both triglycerides and LDL-C, as well as a 41% lower risk of CHD (Dewey et al., 2017). Accordingly, evinacumab, the ANGPTL3 inhibitor, has been verified to reduce the level of the triglyceride in both HoFH patients and the healthy (García-Alonso et al., 2020). In a phase III study, 65 patients with HoFH were randomly assigned to evinacumab (*n* = 43, 15 mg/kg, every 4 weeks) or placebo (*n* = 22) (Raal et al., 2020). All patients received stable lipid-lowering therapy. The primary outcome was the change of LDL-C level from baseline to week 24. Ultimately, LDL-C levels decreased by 47.1% in the evinacumab-treated group, however, LDL-C increased by 1.9% in the placebo group.

Inflammatory cytokines actively participate in the pathogenesis and progression of the CVDs and inflammatory cytokines-targeted therapies could be considered for the treatment of CVDs. Previously, interleukin-1 (IL-1) has been indicated to contribute to CVDs via mediating the inflammatory response, and act as an upstream regulator of a series of inflammatory cytokines (Grebe et al., 2018; Schindler et al., 1990). Based on the above findings, several studies have been conducted to investigate the potential efficacy of IL-1 mAbs in CVDs. IL-1 mAbs can be simply divided into two categories according to the mechanisms: 1) Targeting to the IL-1 receptor (IL-1R); 2) Neutralizing the IL-1 protein. As the first IL-1R antagonist (IL-1Ra) approved by the FDA (Correction, 2017), anakinra has been used in several clinical studies (Table 1) for the treatment of cardiac remodeling after MI (Abbate et al., 2015), CHD (Ikonomidis et al., 2014) and decompensated systolic HF, and the safety has been confirmed (Van Tassell et al., 2017; Trankle et al., 2019). However, the short half-life of anakinra requires a daily injection, which remains a main drawback in its clinical application (Kaiser et al., 2012). With regard to the neutralizing mAb, canakinumab presented a good example, which could inhibit IL-1 binding to IL-1R via targeting to the IL-1β (Dhimolea, 2010; Ozdogan and Ugurlu, 2017). Canakinumab has been approved by the FDA to treat cryopyrin-associated periodic syndromes (CAPS) in 2009 (Nelson et al., 2010). A phase III clinical trial recruited patients with prior MI and high-sensitivity C-reactive protein (hsCRP) ≥ 2 mg/L to test the effect of canakinumab to prevent hospitalization for HF (HHF) (Everett et al., 2019). A total of 10,061 patients were randomly assigned to canakinumab 50 (*n* = 2,263), 150 (*n* = 2,284), 300 mg (*n* = 2,170) or placebo (*n* = 3,344), given subcutaneously once every 3 months. During a median follow-up of 3.7 years, 385 patients had the HHF event. Compared with the placebo group, the unadjusted HRs for HHF in different dose group were 1.04 (95% CI, 0.79–1.36) for 50 mg, 0.86 (95% CI, 0.65–1.13) for 150 mg, and 0.76 (95% CI, 0.57–1.01) for 300 mg (*p* = 0.025). Canakinumab could reduce the incidence of HHF in a dose-dependent manner (Everett et al., 2019). Canakinumab was also used to treat Covid-19 infected patients with myocardial injury due to inflammation (Sheng et al., 2020). A total of 45 Covid-19 infected patients with B-type natriuretic peptide (BNP) or NT-proBNP and CRP elevation were randomly assigned to receive canakinumab 600 mg (*n* = 15), canakinumab 300 mg (*n* = 15) or placebo (*n* = 15). The primary endpoint was the time in days from randomization to either discharge from the hospital or an improvement of two points on a seven category ordinal scale. The trial is still in progress. Rilonacept is a soluble IL-1 decoy antibody. Two IL-1 receptor chains extend to the out space of the membrane, fusing to form a “trap” for neutralizing IL-1α or IL-1β (Peiro et al., 2017). In 2008, rilonacept was approved by the FDA to treat CAPS (Hoffman, 2009). At present, a phase III trial uses rilonacept to treat the pericarditis (Klein et al., 2020). The trial enrolled 86 patients with recurrent pericarditis and systemic inflammation (high CRP levels). The trial comprised of four periods: the screening period; a single-blind run-in period during which rilonacept was used in all patients and background pericarditis medications were tapered; a double-blind period during which patients were randomly assigned to rilonacept or placebo; and long-term extension treatment during which suitable patients would receive rilonacept for 24 months. Preliminary results suggested that after the first dose of rilonacept, both reported pain and inflammation were obviously alleviated. Patients receiving rilonacept could wean from all other pericarditis medications without a recurrence (Fernandez-Ruiz, 2020).

**TABLE 1:** Clinical trials of anakinra.

Disease	Stage	Intervention	Primary outcome	Result
STEMI (Abbate et al. (2015))	II	Anakinra: 100 mg/d for 14 days (*n* = 20) or placebo (*n* = 20)	Death, cardiac death, recurrent AMI, stroke, UA, and symptomatic HF	HR: 1.08 (95% CI: 0.31 to 3.74, *p* = 0.90) for the combined end point of UA, recurrent AMI, death, or stroke. HR: 0.16 (95% CI: 0.03 to 0.76, *p* = 0.008) for death or HF
CHD (Ikonomidis et al. (2014))	Cross-over trial	80 patients with RA (60 with CHD and 20 without CHD) were randomly assigned to a single dose of anakinra (100 mg) or placebo. After 48 h, patients were assigned to the alternate treatment (placebo or anakinra)	Changes of (1) flow-mediated dilation of brachial artery; (2) systemic arterial compliance, ejection fraction, coronary flow reserve, and resistance by echocardiography; (3) peak twisting, left ventricular global longitudinal and circumferential strain, untwisting velocity by speckle tracking; (4) malondialdehyde, nitrotyrosine, interleukin-1β, fas/Fas ligand, and protein carbonyl levels	Compared to the non-CHD patients, CHD patients showed a greater improvement of flow-mediated dilation (57 ± 4% vs 47 ± 5%), arterial compliance (20 ± 18% vs 2 ± 17%), ejection fraction (12 ± 5% vs 0.5 ± 5%), coronary flow reserve (37 ± 4% vs 29 ± 2%), resistance (-11 ± 19% vs 9 ± 21%), peak twisting (30 ± 5% vs 12 ± 5%), longitudinal strain (33 ± 5% vs 18 ± 2%), circumferential strain (22 ± 5% vs 13 ± 5%), untwisting velocity (23 ± 5% vs 13 ± 5%), protein carbonyl, apoptotic and oxidative markers (35 ± 20% vs 14 ± 9%) (*p* < 0.01)
HF (Van Tassell et al. (2017))	III	Anakinra short: 100 mg/d for 2 weeks, followed by placebo for 10 weeks (*n* = 20); anakinra long: 100 mg/d for 12 weeks (*n* = 20) or placebo (*n* = 20)	Interval changes in peak oxygen consumption (Vo_2_) and ventilatory efficiency (the VE/Vco_2_ slope)	At week 2, all groups showed no change in peak Vo_2_. At week 12, anakinra long group showed an improvement in Vo_2_ and the VE/Vco_2_ slope

AMI: acute myocardial infarction; CHD: coronary heart disease; CI: confidence interval; HF: heart failure; HR: hazard ratio; PAH: pulmonary arterial hypertension; RA: rheumatoid arthritis; STEMI: ST-segment elevation myocardial infarction; UA: unstable angina

Interleukin-6 (IL-6) has been reported to cause the development and instability of arterial plaques (Yudkin et al., 2000; Boekholdt and Stroes, 2012), participate in ischemia-reperfusion injury (IRI) and increase the mortality of patients with ACS (Sawa et al., 1998; Zamani and et al., 2013). Currently, a phase II clinical trial in which the IL-6 antagonist tocilizumab is used to treat non-ST-segment elevation MI (NSTEMI) has been completed (Kleveland et al., 2016). A total of 117 patients with NSTEMI were randomly designated to receive a single dose of tocilizumab (*n* = 58) or placebo (*n* = 59) before coronary angiography. The primary endpoint was defined as the changes of hsCRP. The results showed that hsCRP in the placebo group was 2.1 times higher than that of tocilizumab (4.2 vs. 2.0 mg/L/h, *p* < 0.001). High sensitivity troponin T in the placebo group was 1.5 times higher than that in the tocilizumab group (234 vs. 159 g/L/h). After 6 months of follow-up, no safety events were detected. The results indicated that tocilizumab was sufficient to lessen the inflammatory response and myocardial injury in NSTEMI patients.

Abciximab, a Fab fragment of chimeric human-mouse mAb 7E3 (Leung, 2004), could bind to the human platelet glycoprotein (GP) IIb/IIIa receptor and inhibit platelet aggregation by blocking the fibrinogen, von Willebrand factor (vWF) and other adhesion molecules (Nakada et al., 2006). It also binds to vitronectin (αVβ3) receptors found in platelets, vascular walls, endothelial cells and smooth muscle cells (Nesic et al., 2020). Abciximab has been reported to improve the survival rate of STEMI patients with percutaneous coronary intervention (Stone et al., 2012). However, it should not be neglected that the agent would increase the risk of bleeding (De Luca et al., 2005).

MAbs can also effectively treat diseases caused by certain abnormal proteins with high specificity. For instance, abciximab targeting to (GP) IIb/IIIa to treat STEMI has shown a good therapeutic effect in the clinic (Tummala and Rai, 2020). Given that the appealing features of mAbs as well as the encouraging findings mentioned above, it is clear that mAbs therapy has provided a promising therapeutic option for the CVDs, and more efforts should be made to further explore and expand its clinical indications. For example, pro-inflammatory macrophages, the critical mediators during the process of atherosclerosis, are supposed to be inhibited by specific mAbs and therefore may help delay the progression of the diseases (Falk et al., 2013).

#### bAbs

Generally, the occurrence and development of the diseases may be driven by different molecules, in which the monotherapy of mAbs is insufficient for treatment and combined therapy is required (Hicklin and Ellis, 2005). However, it remains challenging to evaluate the safety and effectiveness of mAbs alone when used in combination with other agents, (e.g. another kind of mAbs) at the same time (Henricks et al., 2015). While bAbs, which refer to antibodies binding to two different epitopes at the same time, have showed advantages in tackling these problems (Li et al., 2020).

**FIGURE 2:**
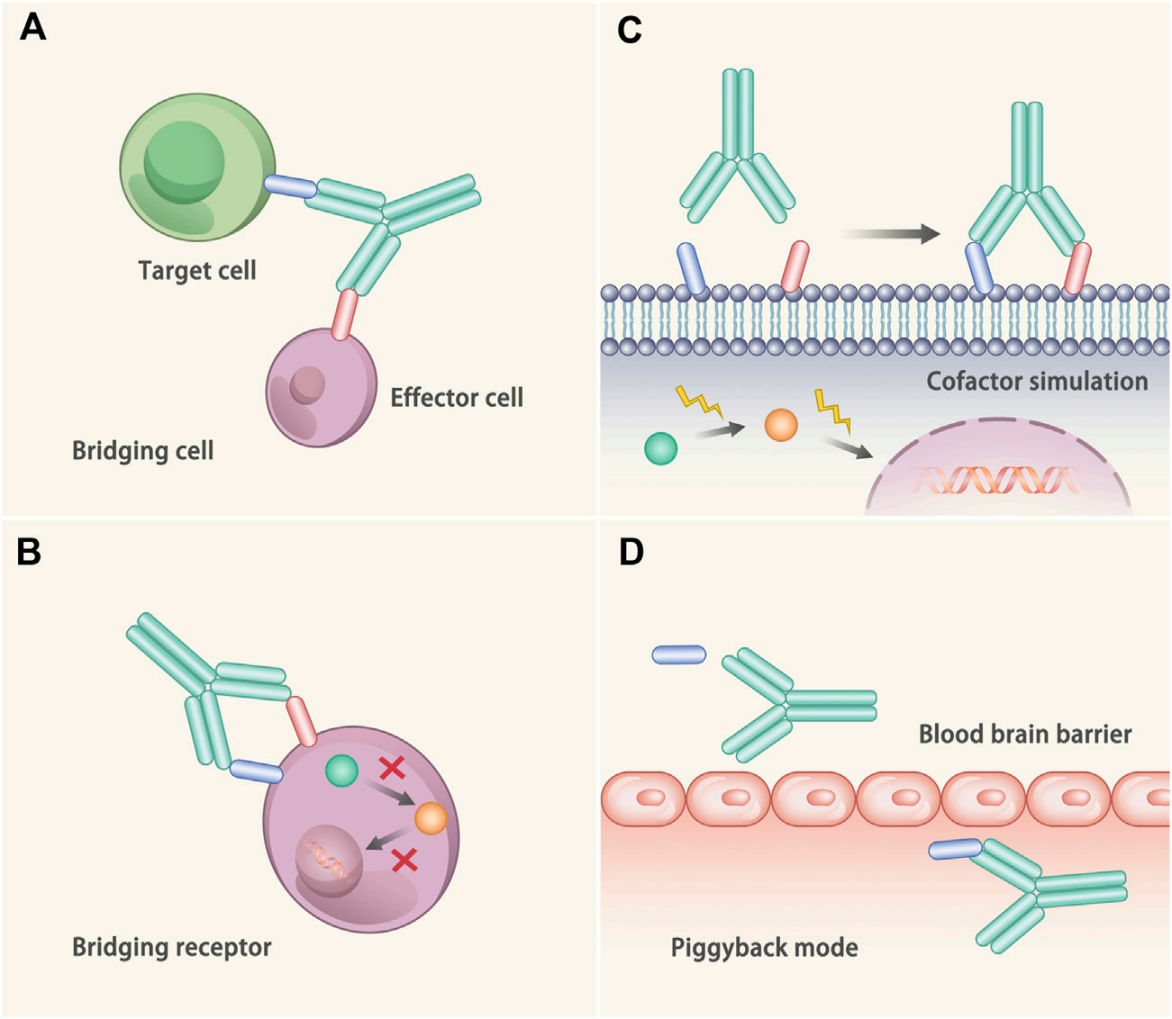
Mechanisms of the bAb. **(A)** Bridging cell. The bAb binds to two different cells at the same time, thus dragging these two cells closer. **(B)** Bridging receptor. The bAb binds to two different proteins on the cell surface and plays a synergistic role, thus inactivating the target cell more efficiently. **(C)** Cofactor simulation. The bAb binds to target antigen and plays the role of agonist to treat diseases. **(D)** Piggyback mode. One of the antigen binding parts of the bAb combines with the target molecule, while the other antigen binding part of the bAb binds to the specific area. In this way, the molecule is transported to the specific area.

The functional mechanisms of bAb to treat diseases mainly includes the following four aspects (Labrijn et al., 2019) (Figure 2). 1) Bridging cells: bAbs bind to the individual binding domains of effector cells and target cells, reducing the distance between the effector cells and the target cells, and promote the targeted-killing efficacy of effector cells. The representative drug is blinatumomab, which binds to CD19 on B cells and CD3 on T cells to treat non-Hodgkin’s lymphoma (Bargou et al., 2008). 2) Bridging receptor: The bAbs bind to two different receptors at the same time to prevent the downstream signal transduction. For example, the epidermal growth factor receptor (EGFR) mAb is widely used to treat tumors, while some tumors can upregulate other receptor tyrosine kinases, such as *MET* proto-oncogene, and develop drug resistance (Engelman et al., 2007). Herein, bAbs against both receptor tyrosine kinases can address the issue. JNJ-61186372 is a bAb that targets to the EGFR and MET, which can inhibit the ligand-induced activation and promote the receptor degradation (Moores et al., 2016). 3) Cofactor simulation: bAbs combine with the target antigens and act as the agonist to treat diseases. One of the representatives is emicizumab, which can bind to FIXa and FX/FXa at the mmol level and remarkably reduce the risk of bleeding in hemophilia A patients (Shima et al., 2016; Kitazawa et al., 2017; Mahlangu et al., 2018). 4) Piggyback mode: One of the antigen binding parts of the bAbs combines with the target molecule, and the other antigen binding part of the bAbs orient to a specific area. In this way, the target molecule will be carried to a certain place. A typical example is the transport of transferrin through the blood-brain barrier (Yu et al., 2011).

bAbs have been used to treat ischemic cardiomyopathy in several studies. One of the obstacles of venous regenerative cell therapy is the low efficiency of cells homing to the target area (Terrovitis et al., 2010; Lalit et al., 2014; Kanelidis et al., 2017). In order to increase the cell homing rate of the myocardial injury region, the researchers developed a bAb, Tand-scFvSca-1 GPIIb/IIIa, which can simultaneously bind to activated platelet GP IIb/IIIa receptor and peripheral blood mononuclear cells (PBMCs) that express stem cell antigen-1 (Sca-1) receptor. Based on that, an IRI mouse model was established and treated with Tand-scFvSca-1 GPIIb/IIIa to increase cells homing to the damaged area. After the treatment, the targeted-PBMCs were successfully transported to the damaged area, and the infiltration of inflammatory cells was significantly reduced (Ziegler et al., 2017). The hematopoietic stem cells (HSCs) possess high plasticity and could differentiate into the nonhematopoietic tissues, which can repair the damaged myocardial tissue in a dose-dependent manner (Zhao et al., 1985; Engelmann et al., 2006; Henning et al., 2007; Vanderheyden et al., 2007; Choi et al., 2011). Therefore, recruiting more HSCs to the ischemic myocardium presents a potential approach to improve the therapeutic effect. Lee et al. synthesized bAbs by chemically coupling anti-CD45 (a common leukocyte antigen identified on HSCs) mAb with myosin light chain (MLC) mAb (Lee et al., 2007). MLC is expressed in myocardial tissue, but only binds to anti-MLC antibodies when the myocardial cell membrane is no longer intact (Lum et al., 2004). Ischemic injury animal models induced by transient ligation of the left anterior descending artery (LAD) were treated with anti-CD45 x anti-MLC bAb (*n* = 9) or phosphate-buffered saline (PBS, *n* = 8). Increased HSCs number in the damaged area and improved cardiac function were observed in animals treated with the bAbs (Lee et al., 2007). Similar to the above principle, bAbs specifically binding to human CD90 and MLC were applied to treat the damaged myocardium (Gundlach et al., 2011). Researchers also exploited bAbs targeting to c-kit expressing on mouse stem cells and VCAM-1, a molecule expressed by injured myocardial cells, to treat the infarcted cardiomyocytes. After ligation of the LAD, animals were injected with anti-c-kit x VCAM-1 (experiment group) or anti-c-kit x isotope control bAbs (control group). The experiment group had more c-kit stem cells homing to the ischemic injury area (Lum et al., 2004). These results indicated that the bAb can reorient stem cells and retain stem cells in the injured myocardium.

In addition to ischemic cardiomyopathy, bAbs are also used to treat vascular lesions. The integrity of the endothelial cells (ECs) plays an essential role in the cardiovascular system. When the integrity of ECs is destroyed, the subendothelial matrix proteins will expose, causing the formation of the platelet-mediated thrombus. GPVI is a soluble platelet collagen receptor (Asahara et al., 1997). CD133 is expressed on the surface of the endothelial progenitor cells (EPCs) (Massberg et al., 2004). BAbs (GPVI-CD133) consisting of soluble platelet collagen receptor GP VI and CD133 mAb were used to treat vascular lesions (Langer et al., 2010). *In vitro*, pig vessels were damaged by a balloon catheter and perfused with EPCs for 2 h. Pig vessels were treated by GPVI-CD133 bAb, GPVI mAb, CD133 mAb, CPVI mAb + CD133 mAb, or PBS. The recruitment of EPCs was evaluated by *in situ* hybridization. Only the GPVI-CD133 bAb group showed increased EPCs recruitment compared to the injury experiments (approximately 10-fold). *In vivo*, GPVI-CD133 bAb enhanced the reendothelialization in mice with the carotid injury. But mice treated by GPVI mAb + CD133 mAb did not show any elevation of EPCs. Therefore, the GPVI-CD133 bAb is a promising treatment for vascular lesions. These results indicated that bAbs could simultaneously bind to two epitopes and realize a “1 + 1 > 2” therapeutic effect.

### Peptides

In the present, peptides are utilized to treat hypertension and vascular diseases. ATRQβ-001, a peptide vaccine made of human angiotensin II (AngII) receptor I and Qβ phage virus-like particles, can inhibit the Ang II-mediated pathway, thus being applied in the treatment of hypertension and aneurysms. In Liao’s et al. studies, the ATRQβ-001 vaccine could effectively decrease the blood pressure in the AngII induced hypertension mice and spontaneous hypertensive rats (SHRs) (Chen et al., 2013). The team also used ATRQβ-001 to treat AngII or calcium phosphate-induced abdominal aortic aneurysm (Zhang et al., 2019). The results showed that ATRQβ-001 could reduce blood pressure and inhibit the expansion of aneurysm and the destruction of the aortic wall in both models. Immunohistochemical analysis confirmed that the vaccine could reduce the infiltration of macrophages and the phenotypic transition of the vascular smooth muscle cell.

ATR12181, the extracellular part of angiotensin receptor 1A (AT1A), is also used to treat SHRs (Zhu et al., 2006). After repeatedly given the vaccine, SHRs produced anti-ATR12181 antibodies, which could attenuate the development of hypertension, diminish the injury of the heart and kidney, and decrease the mRNA levels of c-fos and c-jun in both organs (Li et al., 2014). Meanwhile, ATR12181 vaccine therapy did not cause any autoimmune diseases in the heart and kidney (Zhu et al., 2006). Taken together, the ATR12181 vaccine possesses the potential to treat the hypertension.

Liao’s team also developed the first vaccine against endothelin-1 receptor type A (ETAR) (ETRQβ-002 vaccine) to treat the pulmonary arterial hypertension (PAH) (Dai et al., 2019). In monocrotaline-induced PAH rats and Sugen/hypoxia-induced PAH mice, the ETRQβ-002 vaccine was injected to generate antibodies against ETR-002 (the second extracellular loop of ETAR). The results showed that the ETRQβ-002 vaccine could induce a strong production of anti-ETR-002 antibodies. *In vitro*, the anti-ETR-002 antibodies could attenuate the Ca^2+^-dependent signal transduction induced by endothelin-1. *In vivo*, the ETRQβ-002 vaccine significantly decreased the right ventricular systolic pressure by 10 mmHg in Sugen/hypoxia-induced PAH mice and 20 mmHg in monocrotaline-induced PAH rats. No significant immune injury was observed in the vaccinated animals.

It is worth mentioning that although animal experiments have shown positive effects, clinical trials are needed to test the safety and efficacy of these vaccines.

### Cytokines

Besides using mAbs to antagonize the pro-inflammatory cytokines, researchers also used some cytokines with immunomodulatory function to treat diseases. Regulatory T cells (Tregs) can maintain immune homeostasis and play a crucial role in myocardial repair and atherosclerosis (Ait-Oufella et al., 2006). Interleukin-2 (IL-2) is usually secreted by T cells to stimulate the growth and differentiation of T cells (Li et al., 2001). Studies in preclinical mouse models indicated that IL-2/JES6-1 (IL-2 mAb) complex could increase the number of Tregs and improve the left ventricular ejection fraction (LVEF) after transverse aortic constriction (TAC) (Wang et al., 2016). A randomized, double-blind, dose-escalation, placebo-controlled, phase I/IIa clinical trial is ongoing to assess the tolerability and safety of IL-2 in patients with ischemic heart disease (IHD) and ACS (Zhao et al., 2018b). All patients with IHD and ACS were randomly assigned to receive either IL-2 (0.3–3 × 10^6^ IU) or placebo once daily for 5 days. The study is expected to provide evidence to the immunomodulatory treatment via cytokines in the CVDs.

## Gene Editing Technology

### Clustered Regularly Interspaced Short Palindromic Repeats (CRISPR)/CRISPR Associated Protein 9 (Cas9)

**FIGURE 3:**
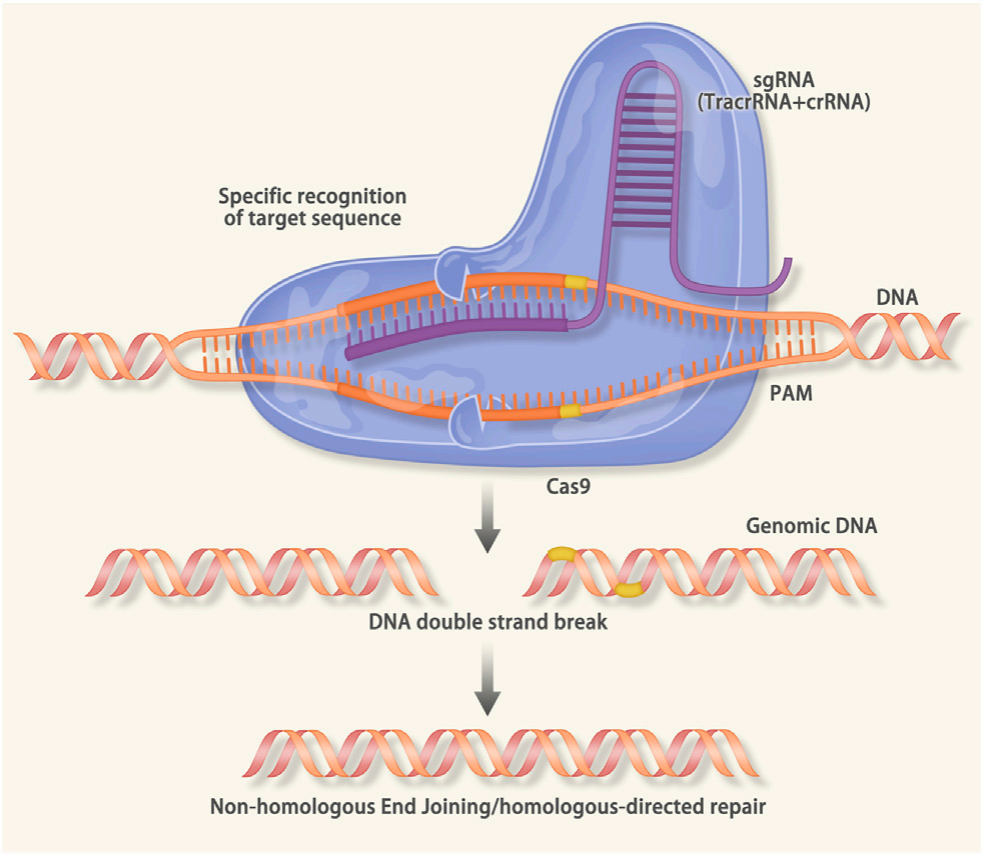
Mechanism of the Clustered Regularly Interspaced Short Palindromic Repeats (CRISPR)/CRISPR associated protein 9 (Cas9): When viruses and foreign DNA invade the host, the cas1 and cas2 protein can recognize the protospacer adjacent motif (PAM) region. The cas1/2 protein will cut the PAM and insert it into the downstream of the leader sequence of *CRISPR*. When the same sequence invades the host, the transcription of precursor CRISPR RNA (pre-crRNA) and trans-activating crRNA (tracrRNA) will be activated. The pre-crRNA, tracrRNA and the cas9 will form a complex that can recognize the sequence that is complementary to the crRNA. After the recognition, the double-strand DNA unwinds to form an R-loop. The crRNA combines with the target sequence via base pairing. Then the double-strand-break (DSB) is induced by the cas9 protease. In the CRISPR/Cas9 gene editing technology, the sgRNA consisting of the tracrRNA and the crRNA is designed *in vitro*. The sgRNA will guide the cas9 to a specific DNA sequence to cause the DSB. After the DSB, endogenous DNA repairs systems (nonhomologous end joining in both dividing and nondividing cells, homology directed repair in the G2/S phase of dividing cells) result in the gene knock-in or knock-out.

CRISPR/Cas9 is associated with immunity against the virus and foreign DNA (Barrangou et al., 2007). It recognizes the protospacer adjacent motif (PAM) region and cleaves the target DNA. Therefore, the expression of exogenous DNA was inhibited (Jinek et al., 2012). CRISPR/Cas9 gene-editing technology uses the artificial small guide RNA to guide the Cas9 protease to break the DNA. After the double-strand break (DSB), gene repair will cause gene knock-out or knock-in and achieve the purpose of modifying DNA. CRISPR/Cas9 is a powerful tool for gene editing, which can accurately edit the gene at a specific point (Figure 3) (Zhang et al., 2014; Xiao-Jie et al., 2015; Savic and Schwank, 2016).

HCM is mainly caused by autosomal dominant inheritance, and there are 1,500 mutations in 15 genes known to cause the disease (Maron et al., 2012). For families with HCM mutations, the gene defects of all fertilized eggs could be corrected by using the CRISPR/Cas9 technology. Currently, CRISPR/Cas9 is used for gene repair at the target sequence. Somatic cells repair the DSB through the non-homologous end joining (NHEJ) mechanism, which leaving a major shortcoming of the off-target effect (Humbert et al., 2012). While the homologous-directed repair mechanism that embryonic cells used in the replication stage is almost error-free (Strong and Musunuru, 2017). Recently, a study used CRISPR/Cas9 to repair HCM gene mutations (Ma et al., 2017). Early injection of a sperm carrying the *MYBPC3* mutation and a CRISPR/Cas9 system resulted in a 100% correction of the mutation.

In addition to HCM, CRISPR/Cas9 has been applied to treat non-ischemic cardiomyopathy. Phospholamban (PLN) participates in the process of the Ca^2+^ homeostasis. The enhanced function of the PLN will impair the heart function (Szymanska et al., 2000). Kaneko et al. knocked out the *PLN* gene of mice with severe HF via CRISPR/Cas9 (Kaneko, 2016). Compared with the control group, mice treated by the CRISPR/Cas9 showed a better cardiac function, a smaller heart size and a higher survival rate. CRISPR/Cas9 also presented advantages in the treatment of other inherited heart diseases. Musunuru et al. injected the CRISPR/Cas9 targeting to the *PCSK9* gene into mice, resulting in loss-of-function mutation of the *PCSK9* gene with a mutation rate of 50%, thus decreasing the plasma PCSK9 concentration, increasing the LDLR expression on the liver surface, and diminishing 35–40% of the plasma cholesterol (Ding et al., 2014). Moreover, no off-target events were detected in the selected 10 loci. Mice carrying H530 R mutation in *PRKAG2* could develop cardiac hypertrophy (Lopez-Sainz et al., 2020). After being corrected by CRISPR/cas9, the heart morphology and the cardiac function of the mutant mice were restored (Xie et al., 2016).

Thyroxine (TTR) amyloidosis is caused by the *TTR* gene mutation and characterized by excessive deposition of thyroxine in the myocardium (Vieira and Saraiva, 2014). Finn et al. employed the CRISPR/cas9 to repair the *TTR* mutation of the mice, resulting in a 97% decrease of plasma abnormal protein levels. Consistent findings were observed in the rat model (Finn et al., 2018). However, whether the method can effectively treat human disease needs further investigation. Duchenne muscular dystrophy (DMD) is a muscular dystrophy caused by a mutation of the dystrophin gene (Falzarano et al., 2015). DMD can lead to dystrophic cardiomyopathy, resulting in high mortality, and there is no effective treatment to slow its progression. Refaey et al. used *mdx* mice (a DMD mouse model) to explore the feasibility of CRISPR/Cas9 for the treatment of DMD (El Refaey et al., 2017). Results showed that the dystrophin protein expression and the cardiac function were restored in the CRISPR/Cas9 treated mice. Long et al. also used CRISPR/Cas9 to treat induced pluripotent stem cells (iPSCs) with duplications, point mutations, or large deletions within the DMD gene (Long et al., 2018). CRISPR/Cas9 successfully restored the dystrophin protein expression in derivative cardiomyocytes.

The CRISPR/Cas9 could also be used to identify whether a gene mutation is pathogenic. The variant of uncertain significance (VUS) is gene mutation for which the pathogenicity is not clear. Ma et al. used CRISPR/Cas9 to analyze an asymptomatic individual carrying mutations in the *MYL3* gene (a cardiomyopathy-associated genetic variant) (Ma et al., 2018). The mutation (NM_000258.2:c.170C > A, NP_000249.1:*p*.Ala57Asp) was shown to be likely pathogenic in the Clinvar database. Patient-derived iPSCs were edited by CRISPR/Cas9 to generate four iPSC lines: 1) “healthy” control without any mutations; 2) homozygous *MYL3* VUS (170C > A); 3) known heterozygous *MYL3* pathogenic mutation (NM_000258.2:c.170C > G); and 4) heterozygous *MYL3* frameshift mutation (170C > A/fs). Only cell lines carrying the known heterozygous *MYL3* pathogenic mutation showed an HCM phenotype at the morphology, gene expression, or functional levels. The above results illustrated the ability of CRISPR/Cas9 to discriminate between benign and pathogenic mutations, thus providing guidance for clinical risk assessment and therapeutic intervention. Other studies showed that CRISPR/Cas9 could be used to identify the VUS that contributed to the CVDs (Garg et al., 2018; Sano et al., 2018).

### Base Editor (BE)

BE is a single base editing technology, which can cause the change from cytosine to thymine using the Cas protein at a specific site (Komor et al., 2016). Initially reported in 2016, the cytidine base editors (CBES) enabled the conversion of cytosine to thymine without causing the DSB (Komor et al., 2016). Researchers have made efforts to test the efficacy of BE in mice (Chadwick et al., 2018). It is well established that loss-of-function mutation of *ANGPTL3* is associated with lower risk of CHD. Musunuru et al. screened the potential gene editing sites using Neuro-2a cells and selected the codon Gln-135 site with the protospacer sequence AGC​CCT​TCA​ACA​CAA​GGT​CA on the *ANGPTL3* gene (Chadwick et al., 2018). Mice were randomly divided into three groups and treated as the following schemes: without sgRNA (BE-control), with sgRNA targeting to *Pcsk9* Trp-159 (BE-*Pcsk9*), with sgRNA targeting to *Angptl3* Gln-135 (BE-*Angptl3*). Primarily, BE-control and BE-*Angptl3* were injected into the C57BL/6J mice. On the day 7, deep sequencing of the liver samples revealed a median editing rate of 35% in the BE-*Angptl3* group, while no gene editing was observed in the BE-control group. Notably, deep sequencing of the top 10 predicted off-target sites demonstrated no evidence of gene editing. Baseline levels of triglycerides, cholesterol, and plasma ANGPTL3 were comparable between the groups, whereas on day 7, significantly lower levels of triglycerides, cholesterol and plasma ANGPTL3 were observed in the BE-*Angptl3* group. Next, the mice were treated with BE-control, BE-*Pcsk9*, BE-*Angptl3*, or a 1:1 mix of BE-*Pcsk9* and BE-*Angptl3*. On day 7, the triglycerides of the BE-*Angptl3* group showed a greater reduction than the BE-*Pcsk9* group. Neither synergism nor additivity was observed in the BE-*Pcsk9* + BE-*Angptl3* group. Finally, the researcher used the *Ldlr*-knockout mice to test the effect of the BE-*Angptl3*. On day 14, compared with the BE-control, BE-*Angptl3* markedly reduced the level of cholesterol (51%) and triglycerides (56%) in treated mice.

Heterozygous T7498C mutation of the *FBN1* gene can lead to the Marfan syndrome (Arbustini et al., 2005). Huang et al. assessed the correction efficacy of BE (Zeng et al., 2018). Cells carrying the FBN1^T7498C^ mutation were transfected with BE and the correctional sgRNA (targeting to the FBN1^T7498C^ mutation). Results showed that 10 of the 20 clones (50%) were edited, and eight clones presented a perfect C to T correction. Subsequently, the experiment was carried out in human embryos. In the testing group, seven embryos were treated by BE and correctional sgRNA. The control group consisted of seven embryos treated by BE and the scrambled sgRNA. Sanger sequencing demonstrated a 100% correction in the testing group.

### CRISPR Interference (CRISPRi) and CRISPR Activation (CRISPRa)

**FIGURE 4:**
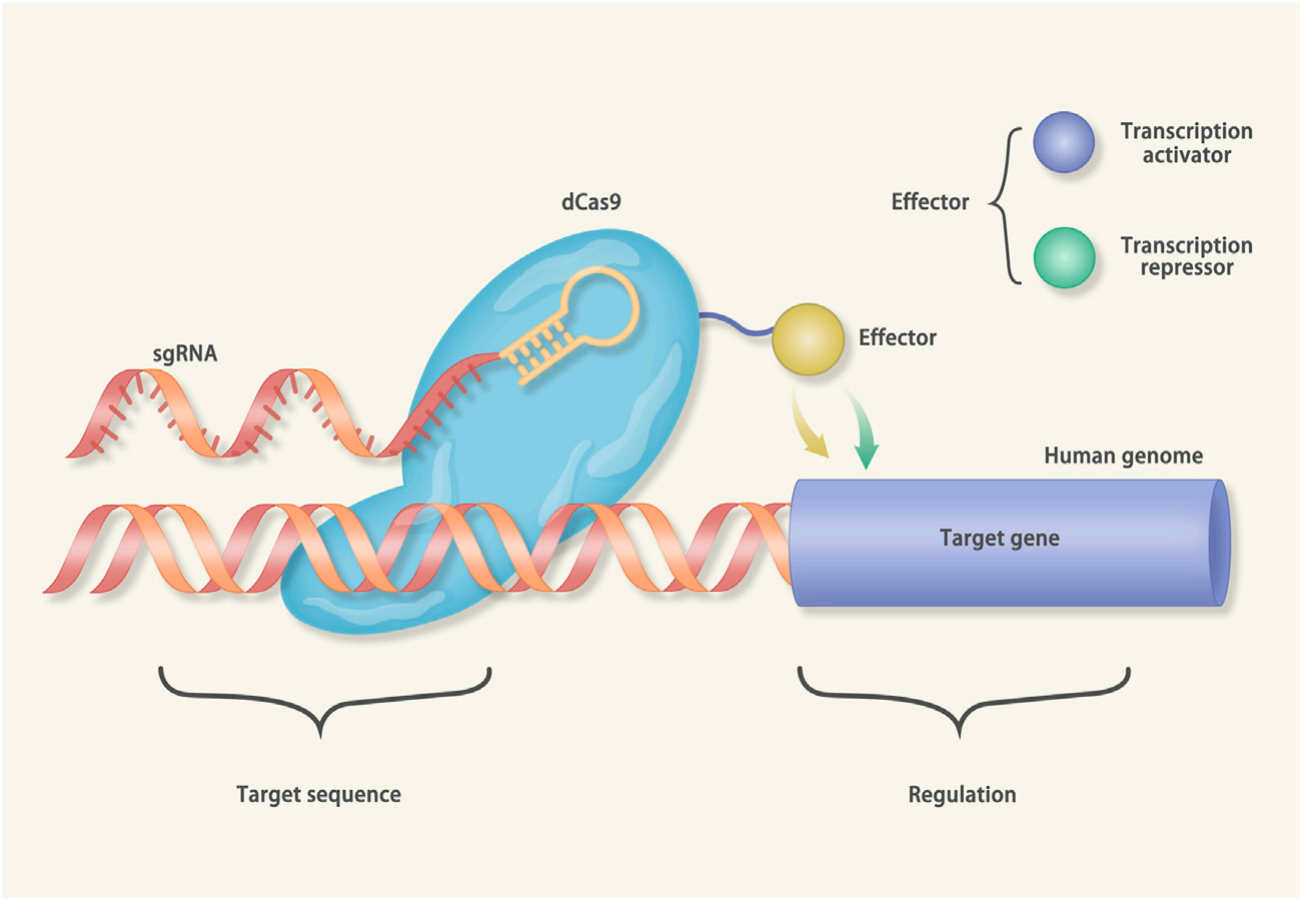
Mechanisms of CRISPR interference (CRISPRi) and CRISPR activation (CRISPRa): The cas protein in the CRISPRi/a is catalytically inactivated (called dcas9). In the CRISPRi, the dcas9 connects with transcriptional suppressors, such as Kruppel associated box (KRAB). Under the guidance of gRNA, the dcas9-KRAB fusion protein binds to the transcription start site (TSS) of the target gene and inhibits transcription. On the contrary, the dcas9 of the CRISPRa is equipped with the transcriptional activator to a given TSS.

Besides editing genes, CRISPR can activate (CRISPRa) or inhibit (CRISPRi) the transcription of the target genes (Zheng et al., 2018). The cas protein used in CRISPRi/a is the “dead” cas9 (dcas9), which is catalytically inactivated. The dcas9 carries an effector molecule (a transcription activator or a transcription repressor) and binds to the target DNA under the guidance of sgRNA (Figure 4). Nowadays, the CRISPRi has been utilized to treat hereditary arrhythmia (Limpitikul et al., 2017). Mutations in calmodulin (*CAM*) can cause long QT syndrome (LQTs) (Crotti et al., 2013; Boczek et al., 2016). The *CAM* gene family consists of three members, *CALM1*, *CALM2*, and *CALM3*. Yue et al. generated iPSCs from a patient harboring a *CALM2* mutation (Limpitikul et al., 2017). The iPSCs were induced to differentiate into cardiomyocytes. The cardiomyocytes exhibited prolonged action potential duration (APD) and mimicked the manifestations of LQTs. In further investigation, after inhibiting the expression of the *CALM2* gene via CRISPRi, the APD of the cardiomyocytes restored to normal. Notably, the CRISPRi only inhibited the expression of the mutant *CALM2* gene while sparing the wild-type counterparts.

Although the CRISPR/Cas9 appears a rather effective gene editing technology, there are still several issues that need to be addressed, such as off-target events (Lanphier et al., 2015). The relatively high incidence of off-target events caused by BE have been proved in previous studies (Zuo et al., 2019). However, *in vivo* and *in vitro* experiments concluded that a careful design of the sgRNA could avoid the off-target events (Akcakaya et al., 2018). Moreover, patients administrated with CRISPR/cas9 may activate the immune response due to the nature of the cas9 as a bacterial protein (Charlesworth et al., 2019). Therefore, it is necessary to consider these factors when applying this technology in clinics.

### Other Gene Editing Technologies

Zinc finger nuclease technology (ZFN) and transcription activator-like effector nucleases technology (TALENs), which consist of the DNA recognition domain and DNA splicing domain, can also be considered as a way for gene editing. ZFN and TALENs have been successfully applied to correct mitochondrial DNA mutations (Bacman et al., 2013; Gammage et al., 2016). According to Gammage’s study, the ZFN could reduce the mutation rate from 73 to 37% in the mice harboring the m.5024C > T tRNA^Ala^ mutation, and greatly improved the cardiac metabolic function of mice (Gammage et al., 2018).

## Nucleic Acid Drugs

The nucleic acid drugs mainly include DNA, microRNA (miRNA), small interfering RNA (siRNA), antisense oligonucleotides (ASO), and mRNA (Wang et al., 2020).

### Vectors for Nucleic Acid Drugs

On the one hand, part of viruses, such as retrovirus, lentivirus, adenovirus and adeno-associated virus (AAV) are commonly used as vectors for nucleic acid drugs (Table 2). Among them, the most widely used virus vector is AAV, which has diverse serotypes with different affinity to tissues (Bacman et al., 2018; Salganik et al., 2015). For instance, AAV9 possesses myocardial tropism (Giles et al., 2018; Wu et al., 2006). On the other hand, the main non-viral vectors (Table 2) include plasmid DNA, lipid nanoparticles (LNP) and N-acetylgalactosamine (GALNAc) (Alabi et al., 2012; Hulot et al., 2016; Springer and Dowdy, 2018).

**TABLE 2:** Gene vectors.

Category	Advantages	Disadvantages
Retrovirus	Retrovirus can effectively integrate into the host genome, and stably express the target genes. The integration mode is transposition, which will not cause genome rearrangement. The transfection efficiency is high	It can only transfer mitotic cells. The host range is narrow. The target gene is small. The virus titer is low. Random integration may lead to activation of oncogene and gene mutation
Adenovirus	A wide range of hosts, high safety and no pathogenicity to human	Low transfection efficiency (10–15%)
Adeno associated virus	A wide range of hosts and no pathogenicity to human. The possibility of insertion mutation is reduced by directional integration. It can stably express the foreign gene	It needs an auxiliary virus to finish the amplification. The preparation process is complex
Lentivirus	Stable expression of target genes, efficient transfection, a wide host range	The virus evolved from HIV-1 and needs to be transformed before use
Plasmid DNA	Easy to produce. No limitation to the DNA size. Low immunogenicity to human	Low transfection efficiency
LNP	Low toxicity, low immunogenicity, biodegradability	Low transfection efficiency; poor stability
GALNAc	High specificity of transportation to liver	Limited application (only binds to cells expressing the asialoglycoprotein receptor)

### DNA

AAV can transfect the cells via the glycosylated cell surface receptors. Following the transfection, AAV will be either transported into the nucleus or degraded by the proteasome. There are two kinds of recombinant AAV: single-stranded AAV (ssAAV) and self-complementary AAV (scAAV) (Wang et al., 2019). The ssAAV should be converted to double-strand DNA before gaining the transcriptional activity, while the scAAV can immediately undergo transcription. Recombinant AAV (rAAV) genomes can be integrated into the host genome at very low frequencies. In addition to virus vectors, plasmid possesses the ability of self-replication and self-expression (Schmeer et al., 2017). Re-expression of the target gene via vectors could provide a strategy for the treatment of the diseases driven by loss-of-function mutation.

The Danon disease caused by loss-of-function of the gene encoding lysosomal associated membrane protein 2 (LAMP2) is a rare X-linked autophagic vacuolar myopathy, which is characterized by multiple system abnormalities, such as heart, skeletal muscle, and liver (Balmer et al., 2005). The penetrance rate of the mutation was almost 100%, and the heart symptoms were extremely serious (D’souza et al., 2014). After the injection of an AAV vector carrying human *LAMP2b* gene, the concentration of LAMP2b protein in heart, liver and skeletal muscle tissue of the *LAMP2* knockout mice notably retrieved, and the symptoms were improved in a dose-dependent manner (Manso et al., 2020). The above findings suggested that the AAV vector could correct the defects via expressing the target gene.

The sarcoplasmic reticulum Ca^2+^-ATPase (SERCA2a) plays a crucial role in the process of Ca^2+^ homeostasis. Besides, decreased activity and expression of SERCA2a were reported in HF patients (Zhihao et al., 2020). In CUPID, a random, double-blind, phase IIb trial, a total of 250 patients with LVEF ≤35% and NYHA class II-IV were enrolled and randomly assigned to receive either placebo (*n* = 127) or AAV1/SERCA2a (*n* = 123) (Greenberg et al., 2016). The primary endpoint was the time from randomization to ambulatory intervention for worsening HF or hospital admission caused by HF. During a median follow-up of 17.5 months, neither improved outcome (HR 0.93, 95% CI 0.53–1.65) nor the safety issues were determined in the AAV1/SERCA2a group. Although the study did not achieve the desired results, it demonstrated the safety of AAV gene therapy.

Proinflammatory cytokines constitute a key part in the pathogenesis of HF (Mann, 2015). Regnase-1 can degrade the mRNA of proinflammatory cytokines. The regnase-1-deficient mice were subjected to TAC to induce HF (Matsushita et al., 2009). Compared to the control littermates, regnase-1-deficient mice showed dilated cardiomyopathy (DCM) and severe inflammation with high level of IL-6 mRNA. Administration of anti-IL6 receptor antibody or AAV carrying the regnase-1 could attenuate the development of cardiomyopathy. Pathological examination proved significant remission of the fibrosis and the infiltration of immune cells (CD45) in the AAV treated mice (Omiya et al., 2020). These results suggested that Regnase-1 carried by AAV could protect the heart by reducing the inflammatory response of cardiomyocytes.

Hypertension can lead to diastolic dysfunction, cardiac remodeling and fibrosis (Tamura et al., 2000; Zile and Brutsaert, 2002). BNP can suppress the renin-angiotensin-aldosterone system (RAAS), thus decreasing the blood pressure (Tamura et al., 2000; Kishimoto et al., 2001). Cataliotti et al. used the AAV9 to continuously enhance the expression of proBNP in the SHR (Cataliotti et al., 2011). A single systemic administration of AAV9 elicited long-term expression of proBNP in the heart, resulting in reductions in diastolic and systolic pressure for 9 months. The posterior wall thickness at end diastole, LV end-systolic dimension, LV mass index and septal wall thickness at end diastole markedly declined, whereas the ejection fraction significantly increased in SHR treated with AAV9-proBNP.

### miRNA

**FIGURE 5:**
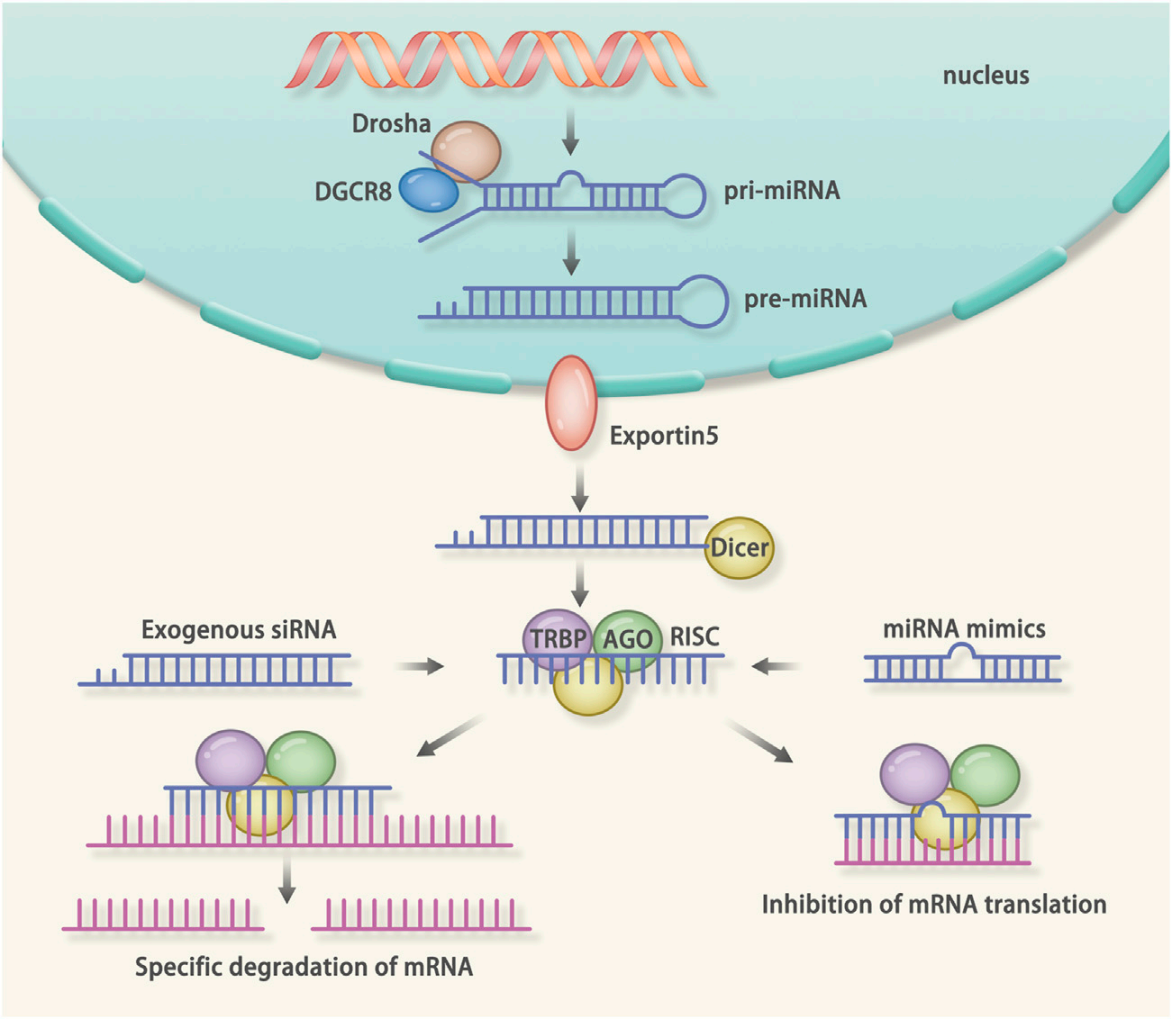
Mechanism of RNA interference (RNAi): RNAi is a post-transcriptional gene silencing method. The microRNA (miRNA) and small interfering RNA (siRNA) can mediate the RNAi. The miRNA is a kind of endogenous non-coding RNA. The miRNA-mediated RNAi starts from the generation of the pri-miRNA. When generated endogenously, the pri-miRNA is cut by the drosha and DGCR8, resulting in the formation of the pre-miRNA. After that, the pre-miRNA is transported into the cytoplasm. Dicer recognizes the pre-miRNA and cuts it into a single strand. Finally, the transactivation response element RNA-binding protein (TRBP), Dicer, Argonaute protein, and the miRNA form the RNA induced silencing complex (RISC). The RISC will bind the complementary mRNA to inhibit the translation. The siRNA is a kind of exogenous non-coding RNA. After delivered into the cells, the siRNA will be cleaved into a single strand RNA. After that, the TRBP, Dicer, Argonaute protein and the siRNA form the RISC. The RISC will degrade the complementary mRNA.

The miRNA is a small endogenous RNA. Most miRNA can inhibit the gene expression via RNA interference (RNAi) (Figure 5) (Wilson and Doudna, 2013). Inversely, some miRNAs activate the transcription, such as miR-589 (Rupaimoole and Slack, 2017). There are two kinds of miRNA drugs: antimiRs and miRNA mimics. The antimiRs primarily target to miRNAs that cause diseases, and the miRNA mimics are designed to inhibit the target mRNA (Janssen et al., 2013). Giacca et al. delivered miR-199 to infarcted myocardium via AAV vector to promote pig myocardial regeneration and realized an obvious diminished infarcted area on day 28 (Gabisonia et al., 2019). Nevertheless, due to the constant expression of the miR-199, the majority of pigs developed arrhythmias, which indicates that the dose of miRNA needs to be strictly controlled. Toll-like receptors (TLRs)-mediated immune responses play an important role in IRI (Linde et al., 2007). It was shown that miR-146a could inhibit TLR-mediated NF-κB signaling pathway (Taganov et al., 2006). Wang et al. constructed lentivirus expressing miR-146a and transfected it into IRI mouse models (Wang et al., 2013). The results showed that miR-146a overexpressing mice had a 55% reduction in myocardial infarct size and maintained a normal ejection fraction. In addition, overexpression of miR-146a inhibited NF-κB signaling pathway and reduced pro-inflammatory cytokine levels. It has been shown that miR-25 can inhibit SERCA2a protein expression, which in turn leads to decreased cardiac function (Jeong et al., 2018). Further *in vivo* experiments showed that overexpression of miR-25 reduced cardiac function in TAC mouse models (Wahlquist et al., 2014). The TAC mouse models treated with anti-miR-25 restored the SERCA2a protein level and cardiac function. However, in SERCA2a-knockout mice, the anti-miR-25 had no effect on the level of SERCA2a and cardiac function (Wahlquist et al., 2014). These results demonstrated that miR-25 affected cardiac function by inhibiting *SERCA2a* and suggested that miR-25 is a therapeutic target for HF. In addition, *in vivo* and *in vitro* experiments showed that miR-92a could inhibit vessel formation and angiogenesis (Bonauer et al., 2009). In MI mouse models, systemic administration of the anti-miR-92a resulted in enhanced blood vessel formation and restoration of the cardiac function (Bonauer et al., 2009). To further validate the therapeutic effect of miR-92a in IHD, Hinkel et al. treated a large animal model of ischemia-reperfusion (pigs) with anti-miR-92a and showed a reduction in miR-92a levels and infarct size (Hinkel et al., 2013).

Currently, a line of preclinical studies focusing on the miRNA therapy are ongoing (Table 3). It should be recognized that RNAi is suitable for the treatment of diseases caused by gain-of-function mutations, but the off-target events largely hampered its application, resulting in no miRNA drugs on the market.

**TABLE 3:** Targeted therapy in cardiovascular disease.

Drug	Disease	Phase
mAb		
evolocumab	High LDL-C hyperlipidemia	On the market Sabatine et al. (2017a)
evinacumab	FoFH	On the market Ahmad et al. (2019)
Anakinra	Heart remodeling, HF	I/II/III Abbate et al. (2008); Abbate et al. (2015); Ikonomidis et al. (2014); Van Tassell et al. (2017); Trankle et al. (2019)
canakinumab	CHD	On the market Ridker et al. (2017)
Rilonacept	Acute pericarditis and atherosclerosis	III/II Dinarello et al. (2012); Klein et al. (2020)
inclacumab	STEMI	II Stahli et al. (2016)
tocilizumab	NSTEMI	II Kleveland et al. (2016)
abciximab	STEMI	On the market De Luca et al. (2005)
bAb		
Sca-1 × GPIIb/IIIa	ICM	Animal model Ziegler et al. (2017)
CD45 × MLC	ICM	Animal model Yu et al. (2015)
c-kit × VCAM-1	ICM	Animal model Lum et al. (2004)
GPVI × CD133	CVD	Animal model Langer et al. (2010)
Peptide		
ATR12181	Hypertension	NDA Zhu et al. (2006)
ATRQβ-001	Hypertension, AAA	NDA Zhang et al. (2019)
siRNA		
Inclisiran	Hypercholesterolemia	NDA Raal et al. (2020)
ASO		
mipomersen	FoFH	III Raal et al. (2010)
volanesorsen	Familial chylomicronemia syndrome	III Witztum et al. (2019)
miRNA		
MRG-110	ICM, HF, PVD	I Gallant-Behm et al. (2018)
miR-33	Atherosclerosis	Animal model Rupaimoole and Slack. (2017)
miR-208	MI	Animal model Montgomery et al. (2011)
miR-21	Cardiac fibrosis	Animal model Thum et al. (2008)
miR-15	MI	Animal model Hullinger et al. (2012)
miR-199	Myocardial regeneration	Animal model Gabisonia et al. (2019)
miR-21	Hypertension	Animal model Alagia and Eritja. (2016)
miR-378	HCM	Animal model Ganesan et al. (2013)
Crispr/cas9	Familial hypercholesterolemia	Animal model Zhao et al. (2020)
	Non ICM	Animal model Kaneko. (2016)
	Myocardial amyloidosis	Animal model Finn et al. (2018)
base editor3	Marfan syndrome	Human embryo Zeng et al. (2018)
CRISPR interference	LQTS	iPSC model Limpitikul et al. (2017)
ZFN	Mitochondrial mutations	Animal model Gammage et al. (2016)
Talen	Mitochondrial mutations	Animal model Bacman et al. (2018)
DNA		
FGF21, AAV: sTGFβR2, AAV:αKlotho	HF	Animal model Davidsohn et al. (2019)
ChR2	Arrhythmia	Animal model Nussinovitch and Gepstein. (2015)
ReaChR	Ventricular tachycardia	Animal model Nyns et al. (2017)
Regnase-1	Heart inflammation, HF	Animal model Omiya et al. (2020)
S16EPLN	Cardiomyopathy, HF	Animal model Hoshijima et al. (2002)
CASQ2	Arrhythmia	Animal model Denegri et al. (2014)
proBNP	Hypertension	Animal model Cataliotti et al. (2011)
betaARKct	HF	Animal model Rengo et al. (2009)
S100A1	Chronic HF	Animal model Pleger et al. (2007)
HO-1	IRI	Animal model Melo et al. (2002)
mRNA		
VEGF-A modRNA	MI	Animal model Zangi et al. (2013)
VEGF-A modRNA	Ischemic complications in type 2 diabetes mellitus	I a/b Gan et al. (2019)
CAR-T	Cardiac fibrosis	Animal model Aghajanian et al. (2019)

AAA, abdominal aortic aneurysm; CHD, coronary heart disease; CVD, cardiovascular disease; FoFH, familial hypercholesterolemia; HCM, hypertrophic cardiomyopathy; HF, heart failure; ICM, ischemia cardiomyopathy; iPSC, induced pluripotent stem cell; IRI, ischemia reperfusion injury; LDL-C, low-density lipoprotein cholesterol; LQTS, long QT syndrome; NDA, new drug application; NSTEMI, non-ST-segment elevation myocardial infarction; PVD, peripheral vascular disease; STEMI, ST-segment elevation myocardial infarction

### siRNA

The siRNA refers to an exogenous double-strand RNA (Kanasty et al., 2013; Nikam and Gore, 2018). After delivered into the cells, the siRNA binds to the complementary sequence to degrade the mRNA (Figure 5) (Wilson and Doudna, 2013). Onpattro, the first FDA approved siRNA drug, targets to TTR and reduces the deposition of amyloid in organs (González-Duarte et al., 2020; Saw and Song, 2020). The Medicines company also developed a type of siRNA drug, inclisiran, to reduce the level of cholesterol via down-regulating the mRNA of PCSK9, thus enhancing the liver's ability to remove LDL-C from the blood (Ray et al., 2017). In addition, there are animal experiments using siRNA to treat myocarditis. Myocarditis can lead to acute HF or chronic DCM (Hua et al., 2020). The level of monocyte chemotactic protein 1 (MCP-1) and its receptor CCR2 were 5-fold higher in patients with myocarditis than in normal controls (Leuschner et al., 2015). Leuschner et al. administered siRNA for CCR2 to treat autoimmune myocarditis mouse models (Leuschner et al., 2015). Mice treated with siRNA not only had an improved ejection fraction, but also showed a reduction in myocardial fibrosis.

### ASO

The ASO can combine with mRNA and inhibit gene expression via the following two manners: 1) RNaseH independent way: After combining with the complementary mRNA, the ASO inhibits translation via the steric blocking effect. 2) RNaseH dependent way: After combining with the complementary mRNA, the ASO recruits the RNaseH to degrade the mRNA (Bennett, 2019).

Some ASO drugs are currently being used in the clinic. For instance, Mipomersen, a synthetic phosphorothioate ASO generated by Genzyme, is proved to down-regulate the mRNA level of apo B-100 (apolipoprotein of LDL and VLDL) via the RNaseH dependent way, thus decreasing the levels of LDL-C, TC and non-HDL-C in patients with familial hypercholesterolemia (Stein and Castanotto, 2017). Familial chylomicronemia syndrome (FCS) is determined as one of risk factors for the atherosclerosis (Benlian et al., 1996). Waylivra (volanesorsen) is a second generation ASO for the treatment of FCS (Witztum et al., 2019). Volanesorsen binds to the 3′ untranslated region of apolipoprotein C-III (apoCIII) mRNA and degrades the apoCIII mRNA via the RNaseH dependent way. A phase III clinical trial enrolled 66 patients and randomly assigned them into volanesorsen group (*n* = 33) or placebo group (*n* = 33) (Witztum et al., 2019). The primary endpoint was the change in fasting triglyceride levels from baseline to the third month. At the third months, an 84% (25.7 mg per deciliter) decrease of plasma apoC-III levels were observed in patients receiving the volanesorsen, whereas a 6.1% (1.9 mg per deciliter) increase of plasma apoC-III levels were detected in patients receiving the placebo. A variety of ASOs are currently available for the treatment of DMD, such as casimersen, golodirsen and eteplirsen (Rodrigues and Yokota, 2018). These drugs use exon skipping technology, which is capable of skipping (deleting) mutated exons, thus avoiding abnormal mRNA splicing and resulting in the synthesis of truncated but partially functional proteins. Casimersen, golodirsen and eteplirsen skip exon 45, 53 and 51, respectively.

### mRNA

The mRNA can be directly translated into proteins. At present, the application of mRNA therapy includes two aspects: 1) Introducing exogenous mRNA into the body and correcting the deficiency of gene expression. 2) Loading the mRNA into the vaccine.

To date, some mRNAs have been developed to treat CVDs. Chien et al. successfully treated the MI mice by using chemically modified mRNA (modRNA) encoding VEGF-A (Zangi et al., 2013). Based on that, Gan’s team evaluated the effects of modRNA encoding VEGF-A in type 2 diabetes mellitus (T2DM) patients (Gan et al., 2019). The trial recruited 42 T2DM patients with the body weight >50 kg and a BMI of 20–35 kg/m^2^. The primary endpoint was to evaluate the tolerability and safety of the modRNA. Compared with the control group (saline), the VEGF-A protein level in the modRNA group was significantly higher. Laser Doppler measurement and acetylcholine iontophoresis suggested that the skin blood flow of the modRNA group increased 2 times. These results indicated that modRNA encoding the VEGF-A could promote vasodilation and neovascularization in T2DM patients. This study supported evidence for the potential that mRNA can effectively treat ischemic symptoms in patients with ischemic CVD and T2DM.

These results show that the mRNA plays an important role in the diseases caused by loss-of-function mutation.

## CAR-T Therapy

**FIGURE 6:**
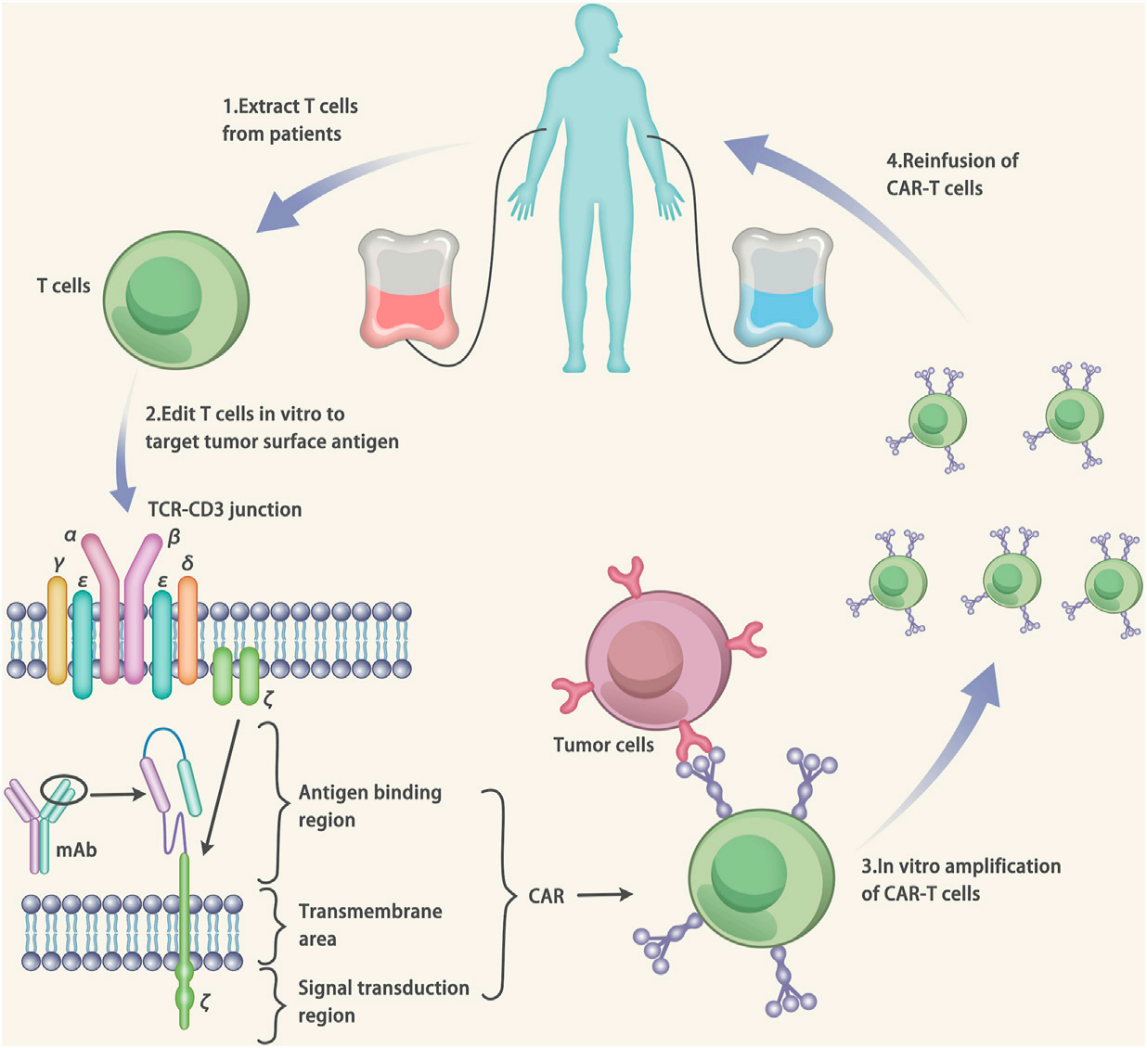
Mechanisms of Chimeric antigen receptor T-cell (CAR-T) therapy. The CAR is made up of three components: 1) an antigen binding region, which consists of a single-chain fragment variable (scFv). The scFv can specifically target to the antigens. 2) the transmembrane area, which fixes the scFv on the surface of T cells. 3) signal transduction region, which consists of CD3-ζ chain of the T cell. The gene of the CAR is designed based on the target antigen. After that, the T cells were extracted from the patients and transfected by vectors carrying the CAR gene. The transfection results in the expression of the CAR on the surface of T cells (CAR-T cells). The CAR-T cells are amplified *in vitro* and injected into the patients to cure the disease.

The CAR-T therapy belongs to immune therapy. The CAR is composed of three parts: 1) An antigen binding region, which consists of a single-chain fragment variable (scFv) and can specifically bind to the target antigens; 2) The transmembrane area, which fixs the scFv on the surface of T cells; 3) Signal transduction region, which consists of CD3-ζ chain of the T cell. When the scFv binds to the target antigen, the CAR-T cells will be activated via the major histocompatibility complex-independent way. (Figure 6) (Depil et al., 2020).

The CAR-T therapy has successfully treated myocardial fibrosis in mice (Aghajanian et al., 2019). The ovalbumin peptide (OVA) is a marker of the activated fibroblast. Herein, a CAR-T cell targeting to the OVA (CAR-T-OVA) was generated and utilized to treat the myocardial fibrosis in mice. Compared with the control group (saline), the fibrosis level of the CAR-T-OVA treated group was extensively alleviated. Besides, the RNA-seq in the cardiomyopathy patients revealed that fibroblast activation protein (FAP) was expressed on the activated fibroblast. Another CAR-T cell targeting to the FAP (CAR-T-FAP) was designed and used to treat the cardiac fibrosis. Compared with the control group (saline), the fibrosis level of the CAR-T-FAP treated group was significantly reduced. The encouraging findings in preclinical experiments paved the way for the CAR-T therapy to be applied to humans. However, it should be noted that some studies have shown that fibroblasts play a protective role in the process of heart injury by secreting matrix and crosslinking with surrounding cells (Hampton, 2019). Therefore, complete elimination of activated fibroblasts is of high-risk. In addition, one of the unexpected side effects of CAR-T is the cytokine release syndrome (CRS) (Ganatra et al., 2019). When the CRS occurs, macrophages release a large number of cytokines. The CRS can lead to cardiac tachycardia, hypotension, pulmonary edema and cardiogenic shock. Currently, there are clinical studies using CAR-T to treat post-transplant lymphoproliferative disorders (PTLD) (Dang et al., 2020). A female patient developed PTLD after receiving the heart transplant, but the disease progressed despite chemotherapy. Ultimately, the patient was treated with CAR-T. The patient was eligible for the indication for CAR T‐cell therapy to treat diffuse large B‐cell lymphoma. The PTLD achieved disease clearance as indicated by PET/CT. And the cardiac function did not decrease during the treatment. This is the first time that CAR-T therapy was used in heart transplant patients, and the success of the treatment, with strict indications and close monitoring of adverse reactions, provides valuable advice for CAR-T in the treatment of CVDs.

## Conclusion

Targeted therapy is a promising method to achieve the precision medicine. Nowadays, various technologies accelerate the development of the targeted therapy. The main technologies of the targeted therapy include protein drugs, gene editing technology, nucleic acid drugs and cell therapy (Table 4).

**TABLE 4:** The advantages and disadvantages of the targeted therapy.

Category	Advantages	Disadvantages
mAb	High specificity; mature clinical application; no off-target events	High price; immune response (except whole human antibody); complex preparation procedures
bAb	High specificity; synergistic effect of different antigen binding domains; no off-target events	Complex preparation procedures; no clinical products; high price
CRISPR/cas9	Specific gene editing	Off-target events; gene rearrangement; oncogenes activation; immune response of the host
BE	Specific gene editing; no gene rearrangement	Low efficiency of gene editing (40%); immune response of the host; off-target events
Nucleic acid drugs	Easy to prepare	Off-target events (miRNA and siRNA); gene insertion (DNA); oncogenes activation
CAR-T	High specificity; no off-target events	Ineffective for intracellular lesions; cytokine release syndrome; complex preparation procedures

The protein drugs consist of mAbs, bAbs and peptides. At first, the mAb is generated from the mouse hybridoma, which carries the heteroantigens and induces the human anti-mouse antibody response (Puligedda et al., 2019). The side effect affects the function of the mAb. The researchers used the transgenic mice, hybridoma, phage display, and single B-cell isolation approaches to design the fully human mAbs, which could avoid the heterogeneous immune response (Jin et al., 2017). The mAbs are of high purity and specificity and can be produced in considerable number quickly. The mAbs have been widely used in the field of tumor and rheumatic diseases. And the mAbs are increasingly applied in the field of CVDs. For example, a series of clinical trials (such as the ODYSSEY OUTCOMES (Schwartz et al., 2018) and the FOURIER trials (Sabatine et al., 2017b)) have confirmed that the PCSK9 mAbs could reduce the cholesterol levels and the mortality of cardiovascular patients. At present, several PCSK9 mAbs have been approved for use in clinics (such as the alirocumab and the evolocumab) (Sabatine et al., 2017b; Schwartz et al., 2018). Although significant achievements have been made in the treatment of CVDs, the preparation process of mAb is complex, which leads to its high price (Carbonetti et al., 2017). Compared with the mAbs, the bAbs show superior advantages of binding to two different epitopes at the same time and exert synergistic effect (Chen et al., 2020). To date, the potential of bAbs to guide stem cells homing to the injured area of myocardium and blood vessels has been investigated (Lum et al., 2004; Gundlach et al., 2011). The results demonstrated that the homing rate of the stem cells increased after the bAbs therapy. But all the conclusions came from animal experiments. Therefore, it is necessary to validate the effect of the bAbs in clinical trials. At present, the peptides are made into vaccines to treat the CVDs. The proteins of the RAAS were designed as vaccine to induce antibody production and inhibit the occurrence of hypertension (Chen et al., 2013; Zhang et al., 2019). In both mouse and rat models, the vaccine has greatly inhibited the elevation of the blood pressure and alleviated organ damage. Similar to the bAbs, the safety and efficacy of the peptides have not been tested in human.

Gene editing technology includes CRISPR/Cas9 technology, ZFN and TALENs, among which the CRISPR/Cas9 is the most widely used. CRISPR/Cas9 can specifically knock out or knock in the target genes (Jinek et al., 2012). Nowadays, researchers use CRISPR/Cas9 to treat HCM, amyloidosis, DCM and cardiac metabolic diseases at the gene level (Maron et al., 2012; Chadwick et al., 2018; Chen et al., 2018). Injection of the sperms carrying the *MYBPC3* mutation and a CRISPR/Cas9 system resulted in a 100% correction of the mutation, indicating the excellent efficiency of CRISPR/Cas9 (Carbonetti et al., 2017). However, the NHEJ mechanism used by the somatic cells to repair the DSB may cause the gene rearrangement, which may lead to the gene dysfunction (Humbert et al., 2012). BE is a variation of the CRISPR/Cas9, which can induce the conversion of the base at the specific site without causing the DSB (Komor et al., 2016). A total of 40% of the mutations in embryos carrying the FBN1^T7498C^ mutation could be corrected via BE (Arbustini et al., 2005). Although the result was not as expected, the research provided innovative concepts for the treatment of inherited CVDs. However, several limitations such as the off-target events and a low success rate confine the clinical application of gene editing technology (Zhan et al., 2019).

Nucleic acid drugs consist of the oligonucleotides, including the DNA, siRNA, miRNA, ASO, and mRNA. Based on the different effects, the nucleic acid drugs can be divided into drugs that promote gene expression (DNA and mRNA) and drugs that inhibit gene expression (siRNA, miRNA, and ASO). The delivery of the nucleic acid drugs into the host necessitates a vector, and the AAV is the most commonly used carrier (Wu et al., 2006; Alabi et al., 2012; Salganik et al., 2015; Hulot et al., 2016; Bacman et al., 2018; Giles et al., 2018; Springer and Dowdy, 2018). For diseases caused by the loss-of-function mutation, a vector carrying the DNA or mRNA can treat the disease by overexpressing the target gene. For diseases caused by the gain-of-function mutation, a vector carrying the siRNA, miRNA or ASO can treat the disease by inhibiting the translation of the target gene. The nucleic acid drugs are easy to produce and relatively cheap. But the foreign DNA may integrate into the host DNA, which may cause the insertion mutation and activation of oncogenes (Wang et al., 2019). Moreover, the RNAi mediated by the siRNA and miRNA may cause the off-target events that inhibit the expression of the non-target gene (Alagia and Eritja, 2016).

After edited *in vitro*, the CAR-T cells can specifically kill the target cells without the MHC restriction (Newick et al., 2017; Zhang et al., 2018). Epstein’s work has proved the excellent efficacy of the CAR-T therapy for the cardiac fibrosis (Aghajanian et al., 2019). Given that the CAR-T cells kill all cells expressing the target antigen, the rigorous design and careful surveillance are indispensable during the application of the CAR-T therapy. However, one of the major shortcomings is that the CAR-T therapy can induce the CRS, which may cause severe heart injury (Ganatra et al., 2019).

In conclusion, targeted therapy has emerged as a novel and promising approach for the treatment of CVDs (Table 3). By utilizing genomics, transcriptomics and proteomics, researchers are capable of dissecting the pathogenesis of the diseases and exploring the target therapy, thus bringing the treatment of CVDs into a precision therapy era. However, the issues of unexpectable off-target events and side effects during the application of the targeted therapies should be addressed in further investigations.
