# Corrigendum: Targeted therapy in cardiovascular disease: A precision therapy era

**DOI:** 10.3389/fphar.2023.1145460

**Published:** 2023-02-03

**Authors:** Mengda Xu, Kailun Zhang, Jiangping Song

**Affiliations:** ^1^ Union Hospital, Tongji Medical College, Huazhong University of Science and Technology, Wuhan, China; ^2^ State Key Laboratory of Cardiovascular Disease, Fuwai Hospital, National Center for Cardiovascular Diseases, Chinese Academy of Medical Sciences and Peking Union Medical College, Beijing, China

**Keywords:** targeted therapy, antibody, gene editing, nucleic acid drugs, cell therapy, cardiovascular disease

In the published article, there was an error in Mengda Xu’s listed **Affiliation**. He is not affiliated with the “State Key Laboratory of Cardiovascular Disease, Fuwai Hospital, National Center for Cardiovascular Diseases, Chinese Academy of Medical Sciences and Peking Union Medical College, Beijing, China,” but rather only with “Union Hospital, Tongji Medical College, Huazhong University of Science and Technology, Wuhan, China.”

In addition, there was an error in the author list, and corresponding author “Kailun Zhang” had not been included. The correct **Author list**, **Affiliation list**, and **Correspondence** information appear below:

“Mengda Xu^1^, Kailun Zhang^1,^* and Jiangping Song^2,^*”

“^1^Union Hospital, Tongji Medical College, Huazhong University of Science and Technology, Wuhan, China, ^2^State Key Laboratory of Cardiovascular Disease, Fuwai Hospital, National Center for Cardiovascular Diseases, Chinese Academy of Medical Sciences and Peking Union Medical College, Beijing, China”

“*Correspondence: Kailun Zhang, whxxprofzhangkl@163.com; Jiangping Song, fwsongjiangping@126.com”

The **Author contributions** section has been revised to reflect this amendment:

“JS and KZ had the idea for the article. MX and JS participated in the literature retrieval. MX contributed to the writing of this review. JS and KZ provided critical appraisal and editorial assistance.”

The authors apologize for these errors and state that this does not change the scientific conclusions of the article in any way. The original article has been updated.

